# Differences in the Characteristics and Pathogenicity of *Colletotrichum camelliae* and *C. fructicola* Isolated From the Tea Plant [*Camellia sinensis* (L.) O. Kuntze]

**DOI:** 10.3389/fmicb.2018.03060

**Published:** 2018-12-11

**Authors:** Qinhua Lu, Yuchun Wang, Nana Li, Dejiang Ni, Yajun Yang, Xinchao Wang

**Affiliations:** ^1^Key Laboratory of Tea Biology and Resources Utilization, Ministry of Agriculture and Rural Affairs, National Center for Tea Improvement, Tea Research Institute of Chinese Academy of Agricultural Sciences, Hangzhou, China; ^2^College of Horticulture and Forestry, Huazhong Agricultural University, Wuhan, China

**Keywords:** *Colletotrichum camelliae*, *Colletotrichum fructicola*, pathogenicity, inoculation, PacC/Rim101

## Abstract

*Colletotrichum*, the causative agent of anthracnose, is an important pathogen that invades the tea plant (*Camellia sinensis*). In this study, 38 isolates were obtained from the diseased leaves of tea plants collected in different areas of Zhejiang Province, China. A combination of multigene (ITS, ACT, GAPDH, TUB2, CAL, and GS) and morphology analyses showed that the 38 strains belonged to two different species, namely, *C. camelliae* (CC), and *C. fructicola* (CF). Pathogenicity tests revealed that CC was more invasive than CF. *In vitro* inoculation experiments demonstrated that CC formed acervuli at 72 hpi and developed appressoria on wound edges, but CF did not develop these structures. Under treatment with catechins and caffeine, the growth inhibition rates of CF were remarkably higher than those of CC, indicating that the nonpathogenic species CF was more vulnerable to catechins and caffeine. Growth condition testing indicated that CF grew at a wide temperature range of 15–35°C and that the optimum temperature for CC growth was 25°C. Growth of both CC and CF did not differ between acidic and weakly alkaline environments (pH 5–8), but the growth of CC was significantly reduced at pH values of 9 and 10. Furthermore, the PacC/RIM101 gene, which associated with pathogenicity, was identified from CC and CF genomes, and its expression was suppressed in the hyphae of both species under pH value of 5 and 10, and much lower expression level was detected in CC than that in CF at pH 6. These results indicated that temperature has more important effect than pH for the growth of two *Colletotrichum* species. In conclusion, the inhibition by secondary metabolite is an important reason why the pathogenicity by CC and CF are different to tea plant, although the environmental factors including pH and temperature effect the growth of two *Colletotrichum* species.

## Introduction

Tea, which is produced by new shoots from tea plants [*Camellia sinensis* (L.) O. Kuntze], is one of the most popular nonalcoholic healthy beverages worldwide. The tea plant is an important economic woody crop and is widely planted in some Asian, African and South American countries, such as China, India, Japan, Sri Lanka, and Kenya. The buds and leaves of the tea plants suffer from many kinds of diseases, resulting in loss of revenue (Guo et al., 2014). Anthracnose is one of the main diseases of the tea plant. Survey results have shown that the morbidity associated with anthracnose, the development of which is related to temperature and humidity, is more serious in the southern region than in the northern region of China (Liu et al., 2016). Mutiple *Colletotrichum* species were the pathogen causing anthracnose of tea plant (Wang et al., 2016a). When *Colletotrichum* invades *Ca. sinensis*, bottle-green, watery lesions emerge on the surface of the leaves early in infection, and the scabs enlarge over time. In the final phase of infection, dense tiny black dots called acervuli form on the lesion; the acervuli, which are small asexual fruiting bodies that can produce conidia which facilitate disease transmission, are the origin of disease spread (Liu et al., 2016).

*Colletotrichum* is a large genus of Ascomycete fungi causing anthracnose disease in a wide range of host genera (Cannon et al., 2012; Takahara et al., 2016). In the tea plant, *Colletotrichum* exists as both a pathogen and an endophyte (Liu et al., 2015). Species of the genus *Colletotrichum* have been isolated from *Ca. sinensis* in China, and 17 known species and 1 indistinguishable strain have been identified based on morphological characteristics and multigene molecular phylogeny (Hyde et al., 2009; Cannon et al., 2012), with *C. Camelliae* (CC) and *C. fructicola* (CF) being the dominant pathogens identified based on the highly isolation rate from the main tea region in China and their pathogenicity (Liu et al., 2015; Wang et al., 2016a). The earliest known record of tea anthracnose in the tea plant was chronicled in 1899 by Massee, and this holotype was labeled CC (Willis, 1899). *Glomerella cingulata* f. sp. *camelliae*, which is the proposed causative agent of disease on *Ca. saluenensis* hybrids, is a homonym of CC (Cannon et al., 2012; Weir et al., 2012; Liu et al., 2015). CC and CF is both a vital pathogen and an endophyte of several plants, of which CC was only isolated from *Camellia* in previous study (Wang et al., 2016a). However, knowledge of the detailed traits of CC and CF in the tea plant is limited.

The majority of *Colletotrichum* have adopted a hemibiotrophic infection strategy to invade host plants; in this strategy, fungi initially develop biotrophic hyphae inside the living host, which later transition to necrotrophic secondary mycelia (O’Connell et al., 1985, 2004; Rao and Nandineni, 2017). In addition, the appressoria and conidia (acervuli) are responsible for initiating infection as well as disseminating the disease and are thus important factors impacting the pathogenicity of the fungus (Emmett and Parbery, 1975; Asakura et al., 2006). Moreover, the appressorium is an important specialized structure that penetrates the cuticle to infect the host plant, and it punctures host surfaces though various processes, such as cell wall melanization, glycerol accumulation, mechanical forcing and enzymatic degradation (Deising et al., 2000; O’Connell et al., 2012). The basal region of appressoria contains the penetration pore, from which effectors are secreted to suppress or evade basal resistance (Kleemann et al., 2012). *C. higginsianum, C. graminicola, C. gloeosporioides*, and other *Colletotrichum* species have been used as model organisms for the study of the infection process (O’Connell et al., 2012; Ko et al., 2016), but the infection strategies of CC and CF are unclear. Hence, we have limited information for interpreting the similarities and differences in the infection process of these two organisms.

Pathogenicity is the potential capacity to cause disease in host plants. Different fungal species display different levels of virulence toward their host plant. Wang et al. (2016a) considered CC, *C. aenigma*, *and C. endophytica* to be the fungal species most virulent to *Ca. sinensis* in China. Furthermore, the virulence of different isolates belonging to the same species varied. The growth of fungal hyphae, the differentiation of appressoria, and the development of acervuli are important for fungal virulence. Environmental conditions can affect the growth and biological characteristics of fungi. The germination time and growth rate of a fungus depend on temperature, humidity, and surface nutrients (Pasanen et al., 1991). Temperature is one of the vital factors affecting fungal growth. Growth rates decrease or cease at nonpermissive temperatures. The pH is another critical factor in fungal–host interactions. The environmental pH value can alter the activity of enzymes such as polygalacturonase (PG) and pectatelyase (PL) and consequently alter the virulence of pathogens (Mackenzie et al., 1993). Extracellular pH is controlled by a conserved fungal pH response pathway regulated by PacC/Rim101, which is a zinc finger transcription factor in filamentous fungi, contributing to fungal pathogenicity (Li et al., 2004; Antonin et al., 2010; Ment et al., 2014). Several species of *Colletotrichum*, such as *C. higginsianum* and *C. gloeosporioides*, alkalize the pH of their host (Miyara et al., 2010; O’Connell et al., 2012). In addition, PacC/Rim101 also plays an important role in filamentous fungi due to its regulation of pathogenicity genes under alkaline pH conditions (You et al., 2007; Miyara et al., 2010; Ment et al., 2014). Nonetheless, few reports have described the effect of ambient conditions and PacC/Rim101 regulation in CC and CF isolated from the tea plant.

Tea plants are rich in flavonoids and alkaloid, such as catechins and caffeine, which play roles in plant defense (Kuc, 1995). Several reports have indicated that alkaloids and phenolics can enhance plant resistance during pathogen–host interactions (Ahuja et al., 2012; Araujo et al., 2016). Catechins can inhibit the activity of PG and PL in *C. gloeosporioides* during interactions with avocado fruits and thereby slow the softening of avocado fruits after harvest (Prusky et al., 1988). Biotic stress can increase caffeine content, and plants may use endogenous caffeine to resist pathogens (Kim et al., 2010). However, there is little research on the relationship between secondary metabolites of tea plants and CC and CF.

In the present study, we aimed to identify *Colletotrichum* species isolated from *Ca. sinensis* by morphological characteristics and multigene molecular phylogeny, and then explored the difference in the biological characteristics of the strains, determined the pathogenicity of the different strains of *Colletotrichum*, and preliminarily clarified the reason that the discrepancy occurred.

## Materials and Methods

### Collection and Isolation

Leaves of tea plants were collected from the cities of Hangzhou, Lishui and Shaoxing in Zhejiang Province in China. Disease samples were collected from leaves showing visible anthracnose symptoms. The isolates were obtained by a single spore isolation technique described by Cai et al. (2009). The spores were dislodged from the anthracnose lesions with a sterilized small blade and were suspended in sterilized water. The suspension was distributed onto the surface of potato dextrose agar (PDA) culture medium, followed by incubation at 25°C. Single germinating conidia were picked to new PDA plates, and incubation at 25°C was continued to generate the pure isolates.

### Morphological Characterization

The colony and conidial characteristics were determined using methods described by Cai et al. (2009). Mycelial discs (5 mm diameter) were taken from the margin of 5 days old cultures, transferred to PDA plates, and incubated in the dark at 25°C. Each isolate was measured in triplicate. The colony diameter was measured daily beginning 3 days after inoculation in order to calculate the growth rate (mm/d). The colony characteristics were also recorded. Appressoria were generated using a slide culture technique and were observed on the bottom surface of synthetic nutrient-poor agar (SNA) cultures (Cai et al., 2009; Damm et al., 2009). The shape and size of 30 conidia, conidiophores, and appressoria were measured.

### Molecular Characterization

The isolates were incubated on PDA culture medium at 25°C for 7–10 days. Total genomic DNA was extracted with an Ezup Column Fungi Genomic DNA Purification Kit [Sangon Biotech (Shanghai) Company Limited, Shanghai, China] and was stored at −20°C. The DNA samples were used as the templates for PCR amplification. The ribosomal internal transcribed spacer (ITS), actin (ACT), glyceraldehyde-3-phosphate dehydrogenase (GAPDH), beta-tubulin (TUB2), calmodulin (CAL), and glutamine synthetase (GS) genes were amplified (Table 1). The PCR conditions for ITS were as follows: 4 min at 94°C; followed by 35 cycles of 94°C for 30 s, 55°C for 30 s, and 72°C for 30 s; and 10 min at 72°C. The annealing temperatures differed among the genes; the optimum annealing temperature for each gene follows: ACT: 58°C, CAL: 59°C, GAPDH: 60°C, GS: 52°C and TUB2: 55°C. Each PCR mixture (50 μL) included Premix Taq^TM^ (25 μL) (TaKaRa Biomedical Technology Company Limited, Beijing, China), ddH_2_O (22 μL), each primer (1 μL), and genomic DNA (1 μL). The PCR products were examined with electrophoresis on a 1.0% agarose gel. Sequencing of the PCR products was performed by Shanghai Huagene Biotech Company Limited, Shanghai, China (Wang et al., 2016a).

**Table 1:** Primers used in this study.

Gene	Primer	Sequence (5′→3′)	References
ACT	ACT-512	ATG TGC AAG GCC GGT TTC GC	Carbone and Kohn, 1999
	ACT-783	TAC GAG TCC TTC TGG CCC AT	Carbone and Kohn, 1999
CAL	CL1C	GAA TTC AAG GAG GCC TTC TC	Weir et al., 2012
	CL2C	CTT CTG CAT CAT GAG CTG GAC	Weir et al., 2012
GAPDH	GDF	GCC GTC AAC GAC CCC TTC ATT GA	Templeton et al., 1992
	GDR	GGG TGG AGT CGT ACT TGA GCA TGT	Templeton et al., 1992
GS	GSF1	ATG GCC GAG TAC ATC TGG	Stephenson et al., 1997
	GSR1	GCC GGT GGA GGA ACC GTC G	Stephenson et al., 1997
ITS	ITS-1	CTT GGT CAT TTA GAG GAA GTA A	Gardes and Bruns, 1993
	ITS-4	TCC TCC GCT TAT TGA TAT GC	White et al., 1990
TUB2	T1	AAC ATG CGT GAG ATT GTA AGT	O’Donnell and Cigelnik, 1997
	Bt2b	ACC CTC AGT GTA GTG ACC CTT GGC	Glass and Donaldson, 1995

### Phylogenetic Analysis

The accession numbers of the sequences were obtained from NCBI GenBank and are listed in Table 2. SeqMan and DNAStar were used to generate the consensus sequences from the sequences of the forward and reverse primers. A phylogenetic tree was constructed using multilocus sequence analysis. Bayesian inference (BI) was used to construct the phylogenies using MrBayes v. 3.2. MrModel test v. 2.3 was used to select the best-fit models of nucleotide substitution. The dataset was assembled using MAFFT v.7 and modulated by MEGA v. 6.0. Ambiguously aligned regions were excluded, and all gaps were regarded as missing data. Markov chain Monte Carlo (MCMC) sampling was used to reconstruct the phylogenies in MrBayes v. 3.2. Analyses of 6 MCMC chains based on the full dataset were run for 2 × 10^7^ generations and sampled every 100 generations (Yan et al., 2015; Wang et al., 2016a).

**Table 2:** Isolates of the *Colletotrichum* spp. studied and GenBank accession numbers of the generated sequences.

Species	Accession number	GenBank accession					
		ITS	GAPDH	ACT	TUB2	CAL	GS
*C. camelliae*	ICMP 10643, LF897, LC3667	JX010224	JX009908	JX009540	JX010436	JX009630	JX010119
	ICMP 18542, LF899	JX010223	JX009994	JX009488	JX010429	JX009628	JX010118
	CGMCC3.14925, LC1364^∗^	KJ95508	KJ954782	KJ954363	KJ955230	KJ954634	KJ954932
	LC3006, LF214	KJ955101	KJ954802	KJ954383	KJ955250	KJ954654	KJ954952
	LS_5	MH463798	MH463874	MH463911	MH478597	MH478633	MH463836
	LS_6	MH463799	MH463875	MH463912	MH478598	MH478634	MH463837
	LS_7	MH463800	MH463876	MH463913	MH478599	MH478635	MH463838
	LS_8	MH463801	MH463877	MH463914	MH478600	MH478636	MH463839
	LS_11	MH463802	MH463878	MH463915	MH478601	MH478637	MH463840
	LS_19	MH463803	MH463879	MH463916	MH478602	MH478638	MH463841
	LS_23	MH463804	MH463880	MH463917	MH478603	MH478639	MH463842
	LS_24	MH463805	MH463881	MH463918	MH478604	MH478640	MH463843
	LS_25	MH463806	MH463882	MH463919	MH478605	-	MH463844
	LS_26	MH463807	MH463883	MH463920	MH478606	MH478641	MH463845
	LS_27	MH463808	MH463884	MH463921	MH478607	MH478642	MH463846
	LS_28	MH463809	MH463885	MH463922	MH478608	MH478643	MH463847
	LS_29	MH463810	MH463886	MH463923	MH478609	MH478644	MH463848
	LS_30	MH463811	MH463887	MH463924	MH478610	MH478645	MH463849
	MZ_1	MH463812	MH463888	MH463925	MH478611	MH478646	MH463850
	MZ_2	MH463813	MH463889	MH463926	MH478612	MH478647	MH463851
	MZ_3	MH463814	MH463890	MH463927	-	MH478648	MH463852
	MZ_4	MH463815	MH463891	MH463928	MH478613	MH478649	MH463853
	MZ_5	MH463816	MH463892	MH463929	MH478614	MH478650	MH463854
	MZ_7	MH463818	MH463894	MH463930	MH478616	MH478651	MH463856
	MZ_10	MH463819	MH463895	MH463931	MH478617	MH478652	MH463857
	MZ_12	MH463820	MH463896	MH463932	MH478618	MH478653	MH463858
	MZ_13	MH463821	-	MH463933	-	MH478654	MH463859
	SX_1	MH463822	MH463897	MH463934	MH478619	MH478655	MH463860
	TJ_8	MH463824	MH463899	MH463936	MH478621	MH478657	MH463862
*C. fructicola*	ICMP18581, CBS 130416	JX010165	JX010033	FJ907426	JX010405	FJ917508	JX010095
	ICMP 18646^∗^, CBS 125397 MTCC 10906	JX010173	JX010032	JX009581	JX010409	JX009674	JX010099
	LC3315, LF537	KJ955159	KJ954860	KJ954435	KJ955306	KJ954711	KJ955010
	LC3427, LF649	KJ955191	KJ954892	KJ954464	KJ955338	KJ954742	KJ955041
	LG_1	MH463787	MH463863	MH463900	MH478586	MH478622	MH463825
	LG_2	MH463788	MH463864	MH463901	MH478587	MH478623	MH463826
	LG_3	MH463789	MH463865	MH463902	MH478588	MH478624	MH463827
	LG_4	MH463790	MH463866	MH463903	MH478589	MH478625	MH463828
	LG_5	MH463791	MH463867	MH463904	MH478590	MH478626	MH463829
	LG_6	MH463792	MH463868	MH463905	MH478591	MH478627	MH463830
	LG_7	MH463793	MH463869	MH463906	MH478592	MH478628	MH463831
	LG_8	MH463794	MH463870	MH463907	MH478593	MH478629	MH463832
	LG_10	MH463795	MH463871	MH463908	MH478594	MH478630	MH463833
	LG_14	MH463796	MH463872	MH463909	MH478595	MH478631	MH463834
	LG_17	MH463797	MH463873	MH463910	MH478596	MH478632	MH463835
	MZ_6	MH463817	MH463893	-	MH478615	-	MH463855
	SX_6	MH463823	MH463898	MH463935	MH478620	MH478656	MH463861
*C. xanthorroeae*	BRIP 45094, ICMP 17903, CBS 127831^∗^	JX010261	JX009927	JX009478	JX010448	JX009653	JX010138

*BRIP, Plant Pathology Herbarium, Department of Employment, Economic, Development and Innovation, Queensland, Australia; CBS, Culture collection of the Centraalbureau voor Schimmelcultures, Fungal Biodiversity Centre, Utrecht, The Netherlands; CGMCC, China General Microbiological Culture Collection center, Bejing, China; ICMP, International Collection of Microorganisms from Plants, Auckland, New Zealand; LC, Working collection of Lei Cai, housed at CAS, China; LF, Working collection of Fang Liu, housed at CAS, China; MTCC, Microbial type culture collection and gene bank, India. ^∗^Ex-type culture.*

### Pathogenicity Tests

In this study, the pathogenicity experiments were based on the method described by Wang et al. (2016a). First, healthy, coincident, nonwounded mature tea leaves were washed with tap water and then sterilized in 1% sodium hypochlorite for 3 min. Then, the tea leaves were punctured by sterile needles. Leaves inoculated with a conidial suspension (10^6^ spores/mL) were used as the treated samples, and leaves inoculated with sterile water were used as the controls. The inoculated samples were added to 12 cm plastic Petri dishes and cultured in a growth cabinet at 25°C with a 12/12 h (day/night) photoperiod for 7 days. Finally, Koch’s postulates were employed to investigate the conidia of each strain.

### Plant Material and Light Microscopy

Tea plants (*Ca. sinensis* cv. *Longjing 43*) (LJ43) were obtained from the Tea Research Institute of the Chinese Academy of Agricultural Sciences, Hangzhou, Zhejiang Province, China. Plants infected with the selected strains were grown at 25°C and 90% relative humidity with a 12/12 h photoperiod. Samples were collected at 12, 24, 48, 72, and 96 h postinoculation (hpi).

To visualize fungal colonization on host tissues, samples were destained in ethanol-chloroform (3:1, vol/vol) and stained with 0.025% (wt/vol) aniline blue in lactophenol (O’Connell et al., 2004). For microscopic observation, the samples were mounted on glass slides in 50% glycerol and examined under a Nikon 80*i* microscope (Japan) equipped with a differential interference contrast (DIC) objective.

### *In vitro* Effects on Mycelial Growth in CC and CF

The effects of the tea polyphenols (TP), catechins and caffeine (Aladdin, China) on mycelia growth in CC and CF were assessed using the mycelial growth rate method in solid media (Zhang et al., 2014). The compounds were dissolved in distilled water and then mixed with sterile melting PDA medium to obtain final concentrations ranging from 0.03125 to 16 mg⋅mL^−1^. The PDA medium was poured into 90 mm diameter Petri plates that were then inoculated with 7 mm discs of CC and CF. Each treatment was performed in triplicate, and PDA plates containing distilled water were used as the control. Mycelial growth diameters were measured. The relative inhibition ratio was calculated using the following formula: *I* (%) = [(*C*-*d*)-(*T*-*d*)]/(*C*-*d*) × 100, where *I* (%) is the inhibition, *d* is the diameter of the fungal disc (7 mm), *C* and *T* are the colony diameters of the control and treatment, respectively.

### Environmental Impact on Fungal Growth

The effect of temperature on fungal growth was explored. PDA was poured into 90 mm diameter sterile plates. Hyphae was picked from the slant culture with a sterilized wire loop and inoculated on a PDA plate. The isolates were incubated on PDA for 5 days at 15, 20, 25, 30, 35, and 40°C in the dark. Each treatment was performed in triplicate and was incubated separately. After incubation for 5 days, the colony diameters were recorded (Chavan and Dhutraj, 2017).

The cultural characteristics of the isolates were studied under different pH conditions. Various amounts of hydrochloric acid and sodium hydroxide were used to regulate the pH of the PDA medium. The final pH values of the media were 5, 6, 7, 8, 9, and 10. After the agar solidified, 5 mm discs of *Colletotrichum* strains from the actively growing cultures were removed using a sterile puncher. Each treatment was performed in triplicate, and the plates were incubated at 20°C in the dark for 5 days, after which the colony diameters were recorded (Liu, 2013).

### RNA Extraction, PacC/Rim101 Identification, and qRT-PCR Analysis

Hyphae used in the qRT-PCR experiment were grown in potato dextrose broth (PDB) medium under different pH conditions at 25°C. Total RNA was extracted from hyphae cultivated in PDB medium using the Fungal Total RNA Isolation Kit (Sangon Biotech) according to the manufacturer’s instructions. Total RNA (1 μg) was used in the cDNA reactions with the PrimeScript^TM^ II 1st Strand cDNA Synthesis Kit (TaKaRa). The cDNA products were diluted 10-fold with RNAase-free water before being used as a template for real-time PCR. The reaction mixture contained 5 μL of SYBR Green, 0.8 μL of the forward and reverse primers, 3.2 μL of double-distilled water and 1 μL of template cDNA. The PCR amplification was performed with a LightCycler^®^ 480 system (Roche) according to the manufacturer’s instructions. The PacC/Rim101 gene was identified by OrthoVenn using comparative genome analysis (*E*-value: 1e-10) (Wang et al., 2015). The *C. gloeosporioides* (Cg-14) genome was downloaded from GenBank (accession number SUB133583). CC and CF have been sequenced (unpublished data). The homologous gene of PacC/Rim101 in other *Colletotrichum* species were identified by conducting BLASTP search of PacC/Rim101 gene sequences against the annotated *Colletotrichum* protein sequences. The following primers for PacC/RIM101 were used in the quantitative PCR analysis: 5′-CGATCACCTCAGGACCAGGGGA-3′ and 5′-CGGGGAGGGTTCAAGGGTTGTT-3′. Each experiment was performed in triplicate. For each treatment, one representative set of results is presented as the mean2^−ΔΔCT^value ± SEM.

### Statistical Analysis

The experimental data were subjected to analysis of variance (ANOVA) using the SPSS 18 software (IBM). The mean values were compared by the least significant difference (LSD) method, and differences were considered significant at *P* < 0.05. All of the data are represented as the mean ± SEM of at least three independent experiments.

## Results

### Identification and Characterization of the Isolates

**FIGURE 1:**
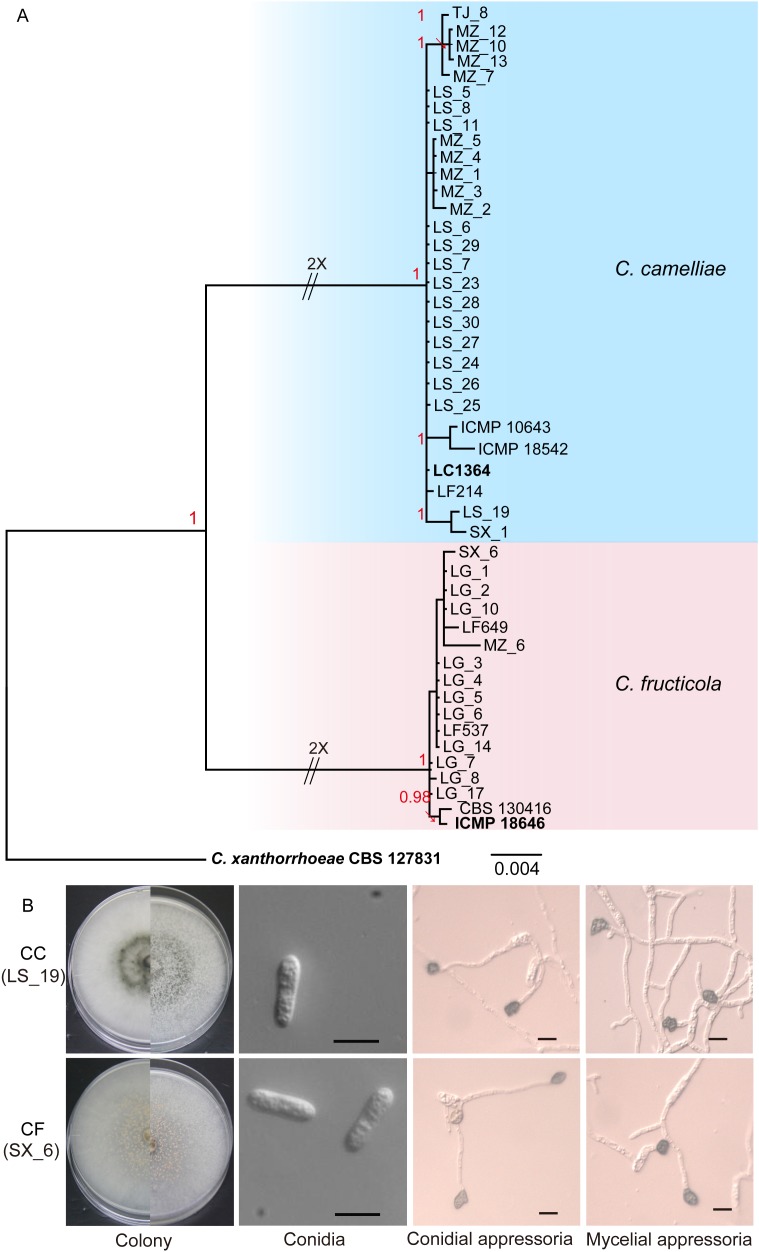
Isolation and identification of *Colletotrichum*. **(A)** Phylogenetic tree generated by Bayesian analysis based on a 6-gene combined dataset (ITS, ACT, GAPDH, TUB2, CAL, and GS) showing the phylogenetic relationships of *Colletotrichum* isolates from *Camellia sinensis*. Bayesian posterior probabilities above 0.95 are shown at each node. *C. xanthorrhoeae* CBS 127831 was used as the outgroup. The scale bar indicates 0.004 expected changes per site. The branches crossed by diagonal lines are shortened by 50%. The ex-type strains are emphasized in bold: LG (Longguan), MZ (Maozhuyuan), and TJ (Tianjian) represent three different sampling points in Hangzhou. LS and SX represent Lishui and Shaoxin, respectively. **(B)** Morphological structures of *C. camelliae* (strain LS_19) and *C. fructicola* (strain SX_6). Scale bar = 10 μm.

In our study, we collected 38 isolates of *Colletotrichum* from tea plants in various regions of Zhejiang Province. Phylogenetic trees (Figure 1A) were constructed using the combined ITS, ACT, GAPDH, TUB2, CAL, and GS gene data that encompassed 47 *Colletotrichum* strains, including *C. xanthorrhoeae* (CBS 127831) as the outgroup. The combined gene alignment, including gaps, comprised 3135 characters. The isolates were found to belong to two subclades, namely, CC and CF. Of our 38 isolates, 25 clustered with the CC ex-type culture (LC1364), and 13 clustered with the type strain of CF (ICMP 18646). The phylogenetic tree had Bayesian posterior probability values ≥ 0.95.

The isolates were cultured on PDA plates and SNA culture medium (in triplicate) for the collection of conidia and appressoria, respectively. Three representative isolates of each species were selected for a survey of their morphological characteristics. On PDA, the CC colonies grew flat with an entire edge with white pigmentation becoming gray toward the center. The pigmentation of the center was gray-white and black on the reverse side; fewer acervuli developed, and these were hidden by the dense mycelia. The CC growth rate was 12.28–13.81 mm/d. On PDA, the CF colonies grew flat with white pigmentation, the reverse side was white, and orange conidial ooze was distributed over the colonies. The CF growth rate was 12.64–13.71 mm/d. The conidial ooze from CF was observed on PDA plates incubated for 3 days at 25°C. However, CC did not produce conidial ooze (Figure 1B and Table 3). We found that the isolates of CF were more capable of producing spores than were the CC isolates.

**Table 3:** Synopsis of morphological data of *Colletotrichum* species.

Strain	Conidia		Appresoria		Growth rate (mm/d)
	Length (μm)	Width (μm)	Length (μm)	Width (μm)	
TJ-8	18.35 ± 0.57	4.95 ± 0.12	9.97 ± 0.12	7.50 ± 0.22	13.81 ± 0.20
LS-19	17.24 ± 0.58	5.01 ± 0.09	9.83 ± 0.18	7.09 ± 0.11	13.67 ± 0.33
LS-11	15.98 ± 0.66	5.15 ± 0.10	9.16 ± 0.21	6.86 ± 0.14	12.28 ± 0.05
LG-5	17.56 ± 0.35	4.87 ± 0.08	7.59 ± 0.28	6.40 ± 0.12	12.64 ± 0.17
SX-6	16.51 ± 0.17	4.64 ± 0.07	10.09 ± 0.19	6.85 ± 0.12	13.71 ± 0.30
MZ-6	15.25 ± 0.34	4.92 ± 0.07	9.52 ± 0.24	6.82 ± 0.10	13.04 ± 0.49

*LS_19, LS_11, TJ_8 belong to C. camelliae, SX_6, LG_5, MZ_6 belong to C. fructicola.*

The appearance of the conidia and appressoria of these two species was similar. The conidia were hyaline, aseptate, smooth, and cylindrical with obtuse ends. For both CC and CF, the appressoria generated on SNA were irregularly shaped, crenate, brown to dark brown, and branched. The length-width ratio of the representative isolates LS_19 and SX_6 was the closest to that of their respective ex-type cultures (Liu et al., 2015). LS_19 (a strain of CC) produced conidia measuring 11.7–27.6 × 4.6–6.2 μm (average (av) ± SEM = 17.2 ± 0.6 × 5.0 ± 0.1 μm, *n* = 30) and appressoria measuring 7.2–11.3 × 6.2–8.1 μm (av ± SEM = 9.8 ± 0.2 × 7.1 ± 0.1 μm, *n* = 30). SX_6 (a strain of CF) produced conidia measuring 14.1–18.9 × 3.9–5.6 μm (av ± SEM = 16.5 ± 0.2 × 4.6 ± 0.1 μm, *n* = 30) and appressoria measuring 8.7–12.2 × 5.7–8.6 μm (av ± SEM = 10.0 ± 0.2 × 6.9 ± 0.1 μm, *n* = 30). Therefore, LS_19 and SX_6 were further used as the representatives CC and CF, respectively, for inoculation.

### Pathogenicity Tests on Tea Plant Leaves *in vitro*

**FIGURE 2:**
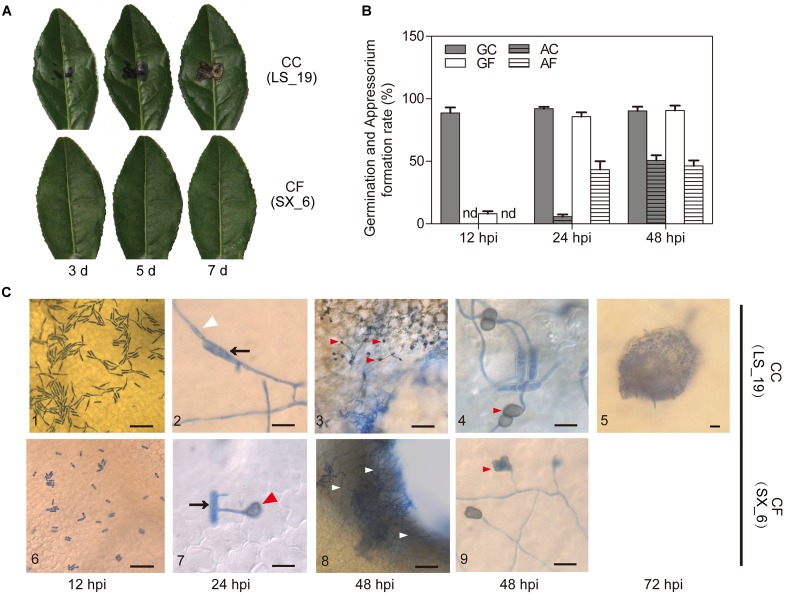
Pathogenicity and inoculation. **(A)** Pathogenicity tests of *C. camelliae* (strain LS_19) and *C. fructicola* (strain SX_6) on tea plant leaves after 3, 5, and 7 days. **(B)** Quantification of germinated conidia and appressoria per 100 conidia. GC, Germination of *C. camelliae*. AC, Appressoria of *C. camelliae*. GF, Germination of *C. fructicola*. AF, Appressoria of *C. fructicola*. nd, not detectable. The error bars represent the SEM; *n* = 100. **(C)** Microscopic examination of fungi stained with aniline blue. C3 and C8 show the wound areas; C4 and C9 show the nonwounded segment. C10 shows an acervul us at 72 hpi. The arrows indicate conidia, the white triangles indicate hyphae, and the red triangles indicate appressoria. Scale bar = 50 μm in C1, C3, C5, C6, and C8; scale bar = 10 μm in C2, C4, C7, and C9.

The pathogenicity of the *Colletotrichum* strains was tested on leaves of tea plants (LJ43) (Figure 2A). Based on pathogenicity studies with tea leaves, significant differences in virulence were observed between CC (LS_19) and CF (SX_6). The CC produced irregular, brown decay lesions around the wound areas. The necrotic lesions were first observed at 3 days postinoculation (dpi); these lesions enlarged over time, and sporogenous structures emerged at 7 dpi. Nonetheless, the SX_6 did not produce the typical lesions of anthracnose disease during the experiment, while LS_19 produced. Following Koch’s postulates, conidia from the lesions were recultivated on PDA medium, and characteristics of these conidia accorded with the original strains after identification. The results suggested that the interspecies virulence was distinct and that CC was more invasive than CF.

### Observation of CC and CF Infection Progress

To clarify the reasons what were the difference of infection progress of CC and CF, the wounded LJ43 leaves were sprayed with a spore suspension (10^6^ spores/mL) for microscopic observation at 12, 24, 48, and 72 hpi. At 12 hpi, the germination rate of LS_19 and SX_6 was 88.7 and 12%, respectively. Appressoria of both species were first seen at 24 hpi, with an occurrence per 100 conidia of 5.7 for LS_19 and 43.3 for SX_6. Over time, the number of germinated conidia and appressoria in both species became no difference (Figure 2B).

Aniline blue was used to visualize the infection structures both species (Figure 2C). We found that both species could grow on the surface of tea leaves at early stages of inoculation (0–24 hpi). At 48 hpi, LS_19 and SX_6 developed filamentous mycelia. We found that a great quantity of hyphae appeared around the wound areas. Interestingly, SX_6 did not develop appressoria around the wounds. On the other hand, we observed appressoria of both species on intact parts of the tea leaves at 48 hpi. As time progressed, LS_19 developed acervuli, which are reproductive structures that are essential for pathophoresis, whereas SX_6 did not differentiate the analogous structures. As the above result showed, the times of germination and appressoria formation of the two species were clearly divergent at the early stage of inoculation. However, LS_19 developed acervuli, which contributed to lesion enlargement.

The thick cuticle of tea plants impedes penetration by pathogens. Therefore, we believed that infection of the necrotic tissues or wound areas, such as the fringe of the wound areas, was beneficial. This result suggested that LS_19 formed appressoria on wound areas and that this step was critical for its pathogenicity.

### Effects of Compounds on Mycelial Growth *in vitro*

**FIGURE 3:**
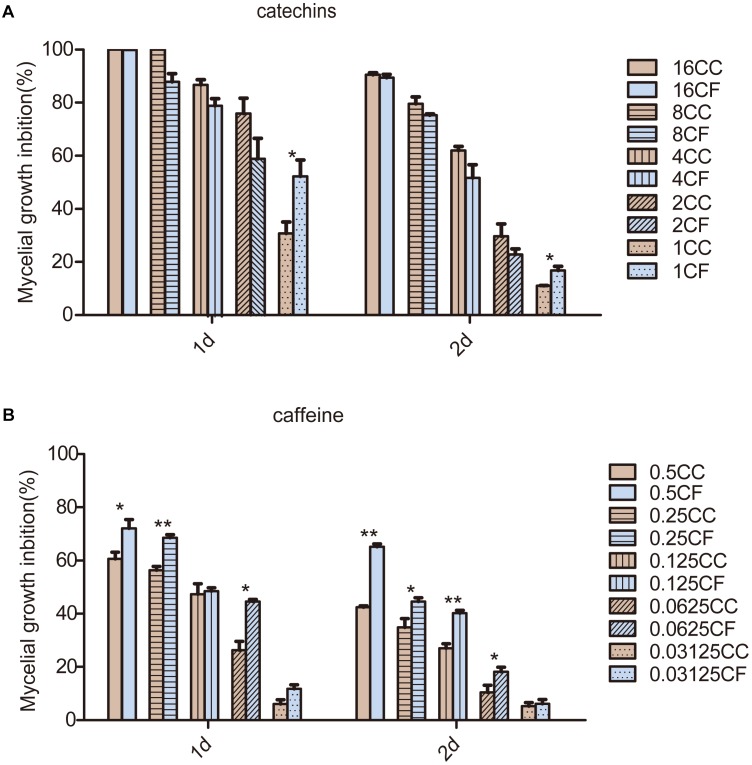
Effects of two compounds on mycelia growth of *C. camelliae* and *C. fructicola in vitro*. **(A)** The mycelial growth inhibition of CC and CF by catechins; **(B)** The mycelia growth inhibition of CC and CF by caffeine. The numbers in the legend represent treatment concentrations (mg⋅mL^−1^); ^∗^*P* < 0.05 and ^∗∗^*P* < 0.01 by the LSD test. The error bars represent SEM; *n* = 3.

We focused on the antifungal activity of caffeine and catechins, which are the most important functional components in tea plants and play a role in plant defense, to evaluate the antifungal activity (Figures 3A,B). The results showed that the inhibition rates of CC and CF were elevated as the concentration of the two compounds increased. Compared with the inhibition rates after 1 day treatment, the antifungal activity decreased after 2 days of treatment. Inhibition rates of CC and CF reached to 100% under the 16 mg⋅mL^−1^ catechins treatment. Inhibition rates of CC and CF exhibit no significant difference at the higher concentrations. However, when the concentration of catechins decreased to 1 mg⋅mL^−1^, the mycelia growth of CF was inhibited to a greater extent than was that of CC. Compared with CC, CF had a higher inhibition rates when fungi were treated with caffeine from 0.0625 to 0.5 mg⋅mL^−1^. Therefore, CF was more vulnerable to catechins and caffeine.

### Effect of Temperature on Mycelial Growth

**FIGURE 4:**
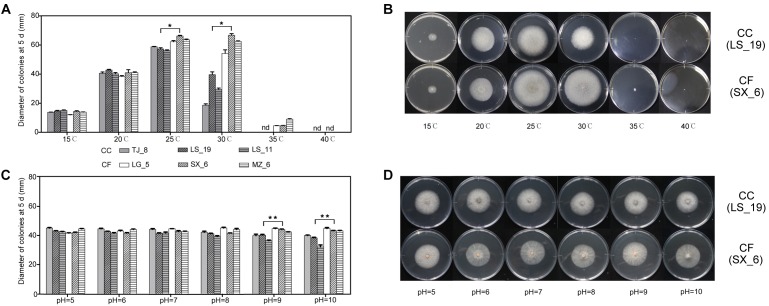
Effect of environmental factors on the growth of *C. camelliae* and *C. fructicola*. **(A,B)** Effect of temperature. **(C,D)** Effect of pH. ^∗^*P* < 0.05 and ^∗∗^*P* < 0.01 by the LSD test (comparing LS_19 and SX_6); nd, not detectable. The error bars represent SEM; *n* = 3.

To test the effect of temperature on the CC and CF mycelial growth, both three strains of these two fungi were grown on PDA medium at different temperatures (Figures 4A,B). The diameter of the CC colonies reached a maximum at 25°C and significantly exceeded that at all other temperatures. The minimum diameter resulted at 15°C. At 30°C, the intraspecific variations in the mycelial growth of the CC colonies were significant. The colony growth was arrested at temperatures higher than 35°C. The maximal diameter of CF occurred at 25 and 30°C. The CF colony diameters at 25 and 30°C were not significant difference, except for that of LG_5. Colony growth continued slowly at 35°C, at which temperature the minimum diameter was observed, and growth was arrested at 40°C. These results illustrated that CC was sensitive to temperature change and that CF grew in a wide permissive temperature range.

The growth characteristics of the typical CC and CF strains LS_19 and SX_6 at different temperatures were compared. The growth rate was not significantly different between LS_19 and SX_6 at low temperatures (15 and 20°C). The SX_6 isolate was the fastest-growing isolate at 25 and 30°C, at which temperatures its diameter was significantly larger than that of LS_19.

### Effect of pH Condition on Mycelial Growth

These six strains were grown on media at different pH values (pH = 5, 6, 7, 8, 9, and 10) at 20^o^C to explore the effects of the acid and base environments on colony growth (Figures 4C,D). The fungi could grow over the tested pH range. Each strain had a slightly different response to each pH except pH 9 and 10, at which the growth of CC was significantly slowed. Thus, the growth of CC was inhibited under a strong alkaline environment. The diameter of CF surpassed that of CC at pH 9. With increasing of pH, the difference in diameter between CC and CF increased. The diameter of LS_19 was lower under a strong alkaline environment. However, SX_6 responded satisfactorily to alternative pH conditions; this phenomenon was also observed for the other CC and CF strains. Both species grew normally in weakly alkaline environments. From the effects of these temperatures and pH conditions on growth, we suggested that CC was more sensitive to environmental change than was CF.

**FIGURE 5:**
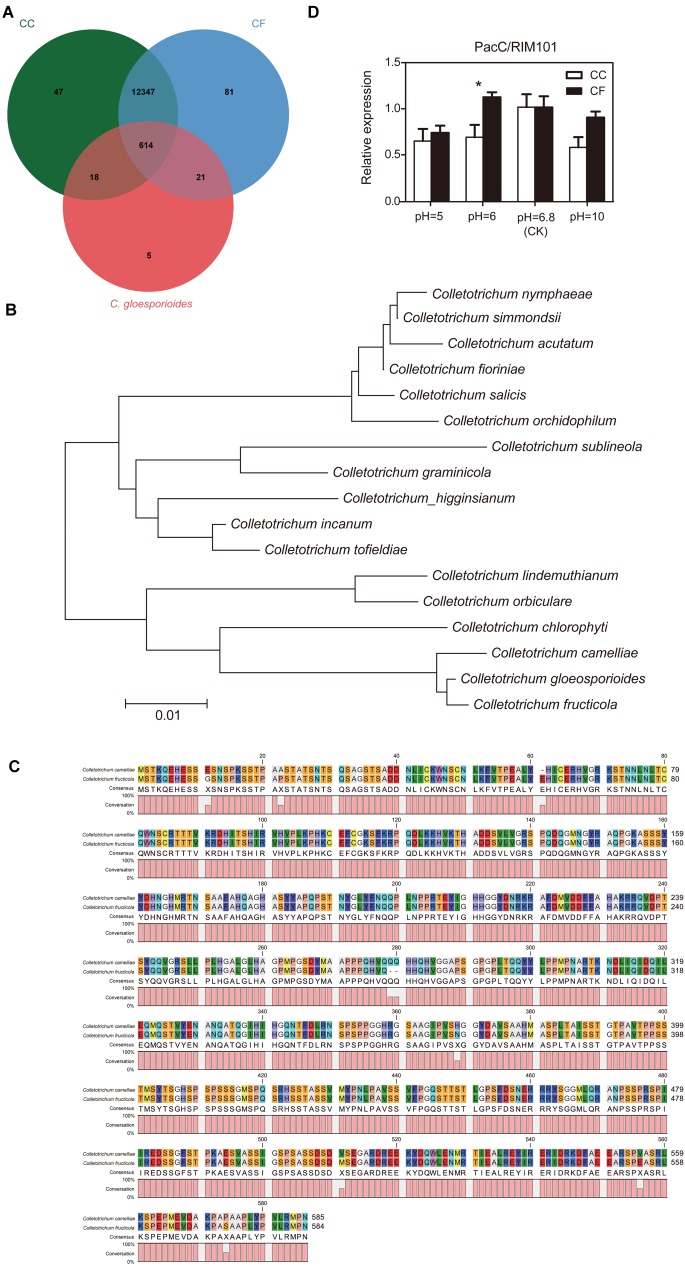
Analysis of PacC/RIM101 expression in *C. camelliae* and *C. fructicola*. **(A)** Venn diagram of the genome summarizing the numbers of sequences among the three species. CC, *C. camelliae*. CF, *C. fructicola.* **(B)** Phylogenetic tree relating PacC/RIM101 orthologs. This tree was constructed with MEGA v 5.0 using the neighbor-joining method. **(C)** Sequence alignment of PacC/RIM101 genes using Clustal Omega (https://www.ebi.ac.uk/Tools/msa/clustalo/) and CLC Sequence Viewer 8. **(D)** Quantitative real-time polymerase chain reaction (qRT-PCR) analysis showing the relative expression of the PacC/RIM101 gene presented as the average and SEM. CC was represented by the LS_19 strain; CF was represented by the SX_6 strain.

To confirm the effect of external pH on fungal growth, the PacC/RIM101 was identified based on comparative genome analysis. The reported 961 *PacC* genes in the Cg-14 genome (Alkan et al., 2013) were compared with those in the CC and CF genomes (unpublished data from our lab). The Venn diagram shows that 614 orthologs gene clusters were found among the three species, and one of the orthologs genes was annotated as a pH response regulator protein (Figure 5A). In order to verify the phylogenetic relationship of this obtained PacC/RIM101 gene in *Colletotrichum* genus, an neighbor-joining (NJ) tree was constructed using the protein sequences of 17 representative *Colletotrichum* species, the results showed that the CC, CF and *C. gloeosporioides* were clustered together, as well as belonged to *C. gloeosporioides* species complex (Figure 5B). Among them the identified sequence was translated into putative polypeptides of 585 and 584 amino acids in CC and CF, respectively, and the PacC/RIM101 amino acid sequences of CC and CF have very high homology (98.5% identity) and similar length (Figure 5C). The PacC/RIM101 gene associated with pathogenicity was identified from CC and CF genomes, The expression of this gene *in vitro* was downregulated in the hyphae of both species under pH value of 5 and 10, and lower transcript abundance was detected in CC than that in CF at pH 6 (Figure 5D). Totally, a homologous PacC/RIM101 gene were obtained from CC and CF based on comparative genomes, but the qRT-PCR results suggested that this gene was not associated with CC and CF response to acid base environment *in vitro*.

## Discussion

### Identification and Characterization of the Isolates

Anthracnose, caused by species of the genus *Colletotrichum*, is one of the most important diseases affecting tea plants and leads to economic losses (Guo et al., 2014; Liu et al., 2015). According to previous studies, 17 species of *Colletotrichum* have been reported from tea plants (Liu, 2013; Liu et al., 2015; Wang et al., 2016a). CC and CF were previously proposed to be the dominant species causing anthracnose in tea plants in China (Cai et al., 2009; Guo et al., 2014; Wang et al., 2016a; Liu et al., 2017). In our present research, we isolated 38 *Colletotrichum* strains that belonged to the CC and CF species. In our combined analysis of morphological features and multigene molecular phylogeny, 25 of these isolates clustered with the ex-type culture of CC, and 13 clustered with the type strain of CF. These strains were isolated from three different cities in Zhejiang Province. We obtained only CC in Lishui but found both species in Hangzhou and Shaoxin. Twenty-two of these isolates were collected from Hangzhou. We found only CF in LG region and the majority of isolates in MZ region were CC. The dominant species varied among sampling locations. All isolates had obvious interspecific differences. CC displayed dense mycelia and little sporulation. In contrast, CF, for which orange conidial ooze was observed, possessed a substantial capability to produce spores on PDA culture medium.

### CC Was More Virulent Than CF

Pathogenicity is the potential capacity of a certain species to cause a disease. The primary symptom of *Colletotrichum* infection on tea plants is an irregular dark brown lesion. Initially, the affected tea leaves will develop circular lesions that subsequently continue to expand. Finally, the junction of diseased and healthy tissue forms a shape resembling water spots, and sporadic black specks appear on the surface of the scab (Luo et al., 2010). Previous researchers used various standards to measure the pathogenicity of pathogens. The time to the appearance of the lesion or acervuli is one of the standards for evaluating the virulence of the pathogen (Sangdee et al., 2011). The earlier a fungus generates acervuli, the higher its virulence. In our research, leaves inoculated with CC began to develop lesions 3 dpi, and these spots continued to grow over time. CC first developed acervuli at 72 hpi in our inoculation experiment *in vitro*. However, CF did not develop obvious lesions or acervuli during the experiment. These results showed that CC is highly virulent, and are consistent with those of previous studies (Sharma et al., 2015; Wang et al., 2016a).

Over its long evolutionary history, *Colletotrichum* forms some specialized infection structures. The appressorium is an important factor in the fungus’ ability to invade the host plant (Perfect et al., 1999; O’Connell et al., 2004). In our inoculation experiments *in vitro*, we found that both species formed appressoria on undamaged areas. However, CF did not develop appressoria on wound areas. in the previous reports considered hydrophobicity and surface hardness to be crucial for the initiation of appressoria and that wax monomers could induce appressorium development (Wilson and Talbot, 2009; Ryder and Talbot, 2015). Therefore, we speculated that CC and CF activated discrepant response mechanisms to develop appressoria in wound areas where leaf structures, metabolites and other properties have been changed. Appressoria not only exert physical forces to pierce the host surface but also secrete effectors from the appressorial penetration pores (Irieda and Takano, 2014; Ryder and Talbot, 2015). Virulence effectors in *Colletotrichum* species likely have critical roles in the manipulation of the host plant (Kleemann et al., 2008). In our study, we suggested that the difference in appressorium development between CC and CF leaded to pathogenic variation between these two species.

### Secondary Metabolites Play the Important in Inhibition

During the tea plant – *Colletotrichum* interaction, abundant secondary metabolites in tea plants play a role in plant defense. In the previous study, caffeine provided tea plants significant resistance to anthracnose by impairing mycelia cell walls and plasma membranes (Wang et al., 2016b). Epicatechin can increase the disease resistance of avocado fruit by decreasing the activity of PG, PL and lipoxygenase of *C. gleosporioides* (Prusky et al., 1988; Guetsky et al., 2005). Our results indicated that catechins and caffeine affected the mycelia growth of CC and CF (Figures 3A,B). A difference in inhibition rate between CC and CF was detected only in PDA culture with 1 mg⋅mL^−1^ catechins. Under caffeine treatment, the inhibition rate of CF was significantly higher than that of CC under concentrations of 0.0625–0.5 mg⋅mL^−1^. The inhibition rate of CF was significantly greater than that of CC under the caffeine treatment. Taken together, our results revealed that the growth of CF was more vulnerable that was that of CC to catechins and caffeine. According to above studies, secondary metabolites play the important role in tea plant defense to different *Colletotrichum* species through inhibiting the growth of pathogen.

### Environment Conditions Affect Growth of CC and CF

Environmental condition is one of the vital factors that affect fungal growth. Temperature plays an important role in the development of anthracnose disease through its effects on conidial germination and appressorium development. In previous studies, the optimum temperature of *C. truncatum* isolated from soybean was 25°C, whereas that of *C. siamense*, *C. fioriniae*, and *C. karstii* collected from tea plants was 26–28°C (Liu, 2013; Chavan and Dhutraj, 2017). In the present study, the range of temperatures at which CC could grow and its optimum growth temperature were 15–30°C and 25°C, respectively. The optimal temperature for CF growth is 25–30°C; when temperatures exceed 35°C, mycelium stopped developing. From the effects of temperature, we found that isolates of CC were sensitive to environmental changes, whereas isolates of CF had a wider range of adaptation. Although the CF has stronger adaptability than CC, its pathogenicity was weaker. Hence, we will further clarify the role of temperature on the pathogenesis of these two pathogens.

The pH is another environmental factor that influences fungal growth and infection capacity. In our study, the results showed that both CC and CF can grow normally in environments ranging from acidic to weak alkaline environments. No notable differences in growth were seen between CC and CF between pH 5 and pH 8, but a downward trend in CC was observed when the pH reached 9. When *Colletotrichum* colonizes the surface of the host plant, the alkalization of the surroundings by ammonia is critical for conidial differentiation (You et al., 2007; Miyara et al., 2010; Alkan et al., 2015). When host was attacked by the fungus, the pH of infection site increased to 8 (Yakoby et al., 2000; Prusky et al., 2001). Under pH 8, both of species have no significant difference in growth rate. PacC/Rim101 gene can mediate pH regulation and regulate growth, differentiation, and virulence in some fungi by transcriptional activation or suppression (Peñalva and Arst, H. N. Jr., 2004; You et al., 2007). In our study, we identified PacC/Rim101 gene from Cg-14, CC, and CF genome in order to explore its effects. We found that CF is more closely related to *C. gloeosporioides* than to CC. The relative expression of PacC/Rim101gene in CC and CF had significant difference at 6. With the increasing the pH value, their expression tended to no obvious difference. Therefore, we suggested that the pH value is not the major reason causing the pathogenicity difference between the two pathogens. PacC/Rim101 may be associated with pathogenicity in interaction of tea plant against *Colletotrichum*, but it still needs further study, such as screening for mutants, to proving genes’ function.

The results of this study are important because the widespread persistence of CC and CF on tea plant causes anthracnose to influence plant health. Both these species had prominent interspecific differences. The strains of CC were virulent and were the primary cause of anthracnose. Catechins and caffeine can inhibit CF growth more effectively. CF rarely formed lesions in the pathogenicity test and the *in vitro* inoculation experiments, whereas it had adapted well to varying temperature and acid-base conditions. According to the effect of pH and expression of related gene, infection ability of CC and CF may be less affected by pH changes. In the future, further study is required to explain the mechanism underlying pathogenicity differences between CC and CF.

## Author Contributions

QL, XW, and YY designed the study. QL, YW, and NL performed the experiments. QL, YW, and DN analyzed the data. QL, YW, and XW wrote and revised the manuscript. QL, DN, XW, and YY revised the final version to be published. All authors read the manuscript.

## Conflict of Interest Statement

The authors declare that the research was conducted in the absence of any commercial or financial relationships that could be construed as a potential conflict of interest.
